# Cytogenetic Alterations Observed in Exfoliative Cells of the Tongue and Oral Mucosa of SARS-CoV-2-Vaccinated Patients: Report of Two Cases and a Brief Literature Review

**DOI:** 10.1590/0037-8682-0008-2025

**Published:** 2025-06-02

**Authors:** Lucas Alves da Mota Santana, Michelly Kierkegaard Campos de Oliveira, Maria Vitória Conceição Carvalho, Pedro Henrique Macedo Moura, Marina dos Santos Barreto, Marcos Antônio Lima dos Santos, Pedro Lima dos Santos, Dalmo Correia, Virgínia Kelma dos Santos Silva, Rajiv Gandhi Gopalsamy, Cleverson Luciano Trento, Lysandro Pinto Borges, Wilton Mitsunari Takeshita

**Affiliations:** 1 Universidade Federal de Sergipe, Departmento de Odontologia, Aracaju, SE, Brasil.; 2 Universidade Tiradentes, Departamento de Odontologia, Aracaju, SE, Brasil.; 3 Universidade Federal de Sergipe, Departmento de Farmácia, São Cristóvão, SE, Brasil.; 4 Universidade de São Paulo, Faculdade de Odontologia, Departamento de Estomatologia, São Paulo, SP, Brasil.; 5 Universidade Federal de Sergipe, Departamento de Medicina, Laboratório de Patologia, Aracaju, SE, Brasil.; 6 Universidade Federal de Sergipe, Departmento de Medicina, Aracaju, SE, Brasil.; 7 Universidade Federal de Sergipe, Programa de Pós-Graduação em Ciências da Saúde, Aracaju, SE, Brasil.; 8 Universidade Tiradentes, Departamento de Medicina, Aracaju, SE, Brasil.; 9 Rajagiri College of Social Sciences, Division of Phytochemistry and Drug Design, Department of Biosciences, Kochi, Kerala, India.; 10 Universidade Estadual Paulista Júlio de Mesquita Filho, Faculdade de Odontologia, Departamento de Diagnóstico e Cirurgia, Araçatuba, SP, Brasil.

**Keywords:** Cytogenotoxicity, Oral cavity, SARS-CoV-2

## Abstract

The wide distribution of angiotensin-converting enzyme 2 (ACE2) and transmembrane protease serine 2 (TMPRSS2) in oral tissues, especially in the salivary glands, which are natural reservoirs of severe acute respiratory syndrome coronavirus 2 (SARS-CoV-2), contributes to the classification of the oral cavity as a potential target for the development of lesions. Despite the effective response produced by next-generation immunizers, the possibility of immune escape by new lineages of SARS-CoV-2 cannot be refuted. Therefore, we describe here the occurrence of cytogenetic alterations in orally exfoliated cells of immunized individuals and, based on the literature review, call attention to the need to monitor these cases in the post-pandemic period.

## INTRODUCTION

The coronavirus disease (COVID-19) pandemic has been considered the most sanitary crisis in the recent decades, being directly responsible for a large number of hospitalizations and the collapse of health systems throughout the world^1^. From the first reports of the disease appearing in December 2019 in Wuhan (Hubei province, China) until November 2024, it is estimated that the outbreak caused by severe acute respiratory syndrome coronavirus 2 (SARS-CoV-2), a novel coronavirus type and the main pathogen associated with the emergence of the pandemic, provoked approximately 776.8 million confirmed COVID-19 cases and over 7 million confirmed deaths^2^. Pathophysiologically, patients positive for SARS-CoV-2 showed a broad spectrum of clinical manifestations, including respiratory disorders, cardiac output, kidney failure, and orocutaneous alterations^3^. 

Although the rapid development of immunizers has made it possible to control the disease and end the health emergency, some variants of the novel coronavirus (especially subvariants of Omicron) are potential targets of interest because of their capacity to overcome vaccine-induced immunity and their wide geographical distribution^2^ ^-^ ^4^. Thus, we describe here the occurrence of cytogenetic alterations in the oral cells of vaccinated patients using a micronucleus assay. A review of pertinent literature was conducted using PubMed and SciELO to reinforce the need to monitor these cases during the post-pandemic period. 

## CASE REPORT

In September 2024, two individuals from the same family tested positive for SARS-CoV-2, which was confirmed using a rapid test for COVID-19 IgG/IgM. The clinical manifestations of the infection were mild, with a prevalence of coryza, coughing, and myalgia during 10 days. Clinical data of the patients are presented in Table 1. The medical history revealed that approximately 3three years ago, the patients were infected with the novel coronavirus for the first time, showing a symptomatology similar to that observed currently. To mitigate the symptoms of the disease, dipyrone (1 g) and multivitamin/mineral supplements (B-complex vitamins, zinc, calcium, vitamin D, and vitamin C) were administered, and a period of 5five days away from work activities was recommended. 

**TABLE 1:** General epidemiological data and serological profiles of SARS-CoV-2 vaccinated patients.

Case	Sex	Age	COVID symptoms	Number of doses administered	COVID-19 vaccination schedule*	Orocutaneous manifestations	Saturation level (SpO_2_)	Past medical history	Treatment	Neutralizing antibody levels (%)
# 1	M	53 y	Coryza, cough, and myalgia	4	ChAdOx1 nCoV-19 (Vaxzevria®, Oxford-AstraZeneca), BNT162b2 mRNA (Pfeizer/ BioNTech)	No	98%	Hypertension, Crohn's disease, and benign prostatic hyperplasia	Analgesic and Multivitamin/mineral (MVM) supplement	89,2
# 2	F	54 y	Coryza, cough, myalgia, and ageusia	4	ChAdOx1 nCoV-19 (Vaxzevria®, Oxford-AstraZeneca), BNT162b2 mRNA (Pfeizer/ BioNTech)	No	98%	Fibromyalgia, rheumatism, and arrhythmia	Analgesic and Multivitamin/mineral (MVM) supplement	78

**M:** male; **F:** female; **y:** years; *patients received two doses of each immunizing agent.

**FIGURE 1:**
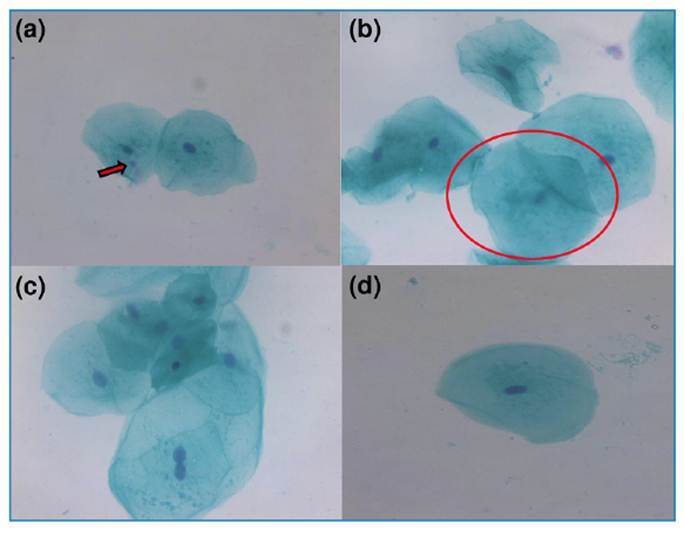
Metanuclear alterations observed in cytological smears of exfoliative cells of the tongue and oral mucosa of SARS-CoV-2 vaccinated patients. **A:** Micronucleus **(red arrow); B:** Karyolysis **(red circle); C:** Binucleation**; D:** Pyknosis. 400× magnification. Feulguen-Fast-Green stain.

Subsequently, patients were investigated for the presence of neutralizing antibodies (Nabs). Laboratory results demonstrated a significant rate of circulating antibodies (89,2% and 78%, respectively; Table 1), which partially explained the mild form of the disease. Moreover, smears of exfoliated cells obtained from the buccal mucosa and tongue of affected individuals were collected using a cytobrush to analyze cytogenetic damage and spread on glass slides. In general, the patients in the present case were non-smokers, no exposure to X-rays was monitored in the last months, and no use of mouthwash or topical medication may have modified the cytological profile of the cells. Subsequently, the oral cells obtained were fixed in a 3:1 methanol/acetic acid buffer solution and stained using the Feulgen/Fast Green method. Slide analysis revealed the presence of cytogenetic alterations, such as micronuclei, karyolysis, binucleation, and pyknosis (Figure 1). 

## DISCUSSION

Despite the World Health Organization (WHO) declaring the end of the COVID-19 pandemic a global health emergency in May 2023, SARS-CoV-2 remains a potential pathogen of interest to public health because of the increasing number of mutations that favor the emergence of new lineages^1^ ^,^ ^4^. Notably, several authors have reported cases of orocutaneous manifestations in vaccinated individuals^3^. Due to the high expression of angiotensin-converting enzyme 2 (ACE2) and transmembrane protease serine 2 (TMPRSS2) in the oral tissues (*e.g*., tongue and mucosa), and because salivary glands are considered reservoirs of SARS-CoV-2, the oral cavity is a potential target for the development of lesions^5^. In addition to changes at the tissue level induced by the virus, such as chronic inflammation and vascular disorders, morphological changes in cellular parameters have also been described^6^ ^,^ ^7^. For instance, Marques *et al*. (2022)^7^ demonstrated that the epithelial cells collected from the dorsum of the tongue of infected patients showed a decrease in perimeter and a smaller nuclear diameter when compared to the control group. 

Another consequence of SARS-CoV-2 infection in oral cells is the possibility of occurrence of mutagenesis and cytotoxicity. In a pioneering study, Pinto *et al*. (2021)^8^ observed an increase in the frequency of micronuclei in cells obtained from the buccal mucosa of patients with COVID-19 as well as a significant rate of cell death. Mutagenesis is associated with the occurrence of micronuclei, and their presence is an important biomarker for the assessment of genomic instability, and consequently, carcinogenesis. On the other hand, cytotoxicity represents the risk of cell death, being characterized by the presence of alterations like karyolysis and karyorrhexis^8^. 

In our study, the main cytogenetic alterations observed were micronuclei formation, karyolysis, binucleation, and pyknosis, as reported by Pinto *et al*. (2021)^8^. However, the mechanisms employed by SARS-CoV-2 in the occurrence of these cytogenetic disorders remain poorly understood. Nevertheless, Da Silva *et al*. (2022)^9^ argue that cell death may occur in response to apoptosis or necrosis through the release of free radicals, mitochondrial dysfunction, and synthesis of inflammatory cytokines, including TNF-α. According to Ren *et al*. (2021)^10^, increase of the frequency of micronuclei in cells with high expression of ACE2 is result of the syncytia formation (*i.e*., cells infected with SARS-CoV-2 fuse with neighboring cells), with subsequent DNA damage accompanied by alteration of intracellular levels of cGAS and γH2Ax. Thus, our preliminary findings call attention to the monitoring of COVID-19 cases in vaccinated patients, who may demonstrate varying degrees of susceptibility. 

Regarding vaccine-induced immunity, several hypotheses have been proposed to explain the different grades of immunization agents^11^ ^,^ ^12^. According to Abou-Saleh *et al*. (2022)^11^,neutralizing antibodies against SARS-CoV-2 produced after mRNA vaccine administration (*e.g*., Pfizer BNT162b2 and Moderna mRNA-1273) declined over time. Moreover, vaccine immunogenicity is reduced in immunocompromised individuals^12^. Notably, the patients mentioned here had chronic diseases, such as hypertension, Crohn's disease, and rheumatism. Anti-inflammatory drugs used to treat complications can generate negative feedback by suppressing vaccine-induced immune responses. Furthermore, the omicron variant has a unique ability to escape the immune response compared with other SARS-CoV-2 variants^4^.

In summary, our findings suggest that SARS-CoV-2 infection can induce significant cytogenetic alterations in the oral tissues of vaccinated patients, highlighting the importance of post-pandemic monitoring. Finally, future rigorous epidemiological studies in large populations should investigate the possible mechanisms underlying SARS-CoV-2-induced cytogenetic alterations, and evaluate the effectiveness of preventive strategies. 
